# Comparison of Marker- and Markerless-Derived Lower Body Three-Dimensional Gait Kinematics in Typically Developing Children

**DOI:** 10.3390/s25144249

**Published:** 2025-07-08

**Authors:** Henrike Greaves, Antonio Eleuteri, Gabor J. Barton, Mark A. Robinson, Karl C. Gibbon, Richard J. Foster

**Affiliations:** 1 Research Institute for Sport and Exercise Sciences, Liverpool John Moores University, Liverpool L3 3AF, UK; henrike.greaves@alderhey.nhs.uk (H.G.); g.j.barton@ljmu.ac.uk (G.J.B.); m.a.robinson@ljmu.ac.uk (M.A.R.); k.c.gibbon@ljmu.ac.uk (K.C.G.); 2 Alder Hey Children’s NHS Foundation Trust, Liverpool L12 2AP, UK; 3 Research & Innovation Department, Liverpool University Hospitals NHS Foundation Trust, Liverpool L7 8YE, UK; antonio.eleuteri@liverpool.ac.uk

**Keywords:** gait analysis, Theia3D, kinematics, markerless motion capture, plug-in gait model

## Abstract

Background: Marker-based motion capture is the current gold standard for three-dimensional (3D) gait analysis. This is a highly technical analysis that is time-consuming, and marker application can trigger anxiety in children. One potential solution is to use markerless camera systems instead. The objective of this study was to compare 3D lower limb gait kinematics in children using both marker-based and markerless motion capture methods. Methods: Ten typically developing children (age 6–13 yrs) completed five barefoot walks at a self-selected speed. A 10-camera marker-based system (Oqus, Qualisys) and a 7-camera markerless system (Miqus, Qualisys) captured synchronised gait data at 85 Hz. Generalised Additive Mixed Models were fitted to the data to identify the random effects of measurement systems, age, and time across the gait cycle. The root-mean-square difference (RMSD) was used to compare the differences between systems. Results: Significant interactions and differences were observed between the marker-based and markerless systems for most joint angles and planes of motion, particularly with regard to time and age. Conclusions: Despite differences across all kinematic profiles, the RMSD in this study was comparable to previously published results. Alternative model definitions and kinematic crosstalk in both systems likely explain the differences. Age differences were not consistent across joint levels, suggesting a larger sample size is required to determine how maturation may affect markerless tracking. Further investigation is required to understand the deviations and differences between systems before implementing markerless technology in a clinical setting.

## 1. Introduction

Many children experience walking impairments due to congenital deformities, developmental disabilities, acquired injuries, or degenerative changes. Therefore, the gait of children is systematically evaluated in clinical gait laboratories and used diagnostically to inform and evaluate the outcomes of clinical interventions, including orthopaedic or neurological interventions, as well as the provision of orthotic devices [1]. Furthermore, assessing gait and functional ambulation is also essential for documenting the extent of gait changes, disease progression, and treatment effectiveness in patients with unknown and rare diseases.

The current gold standard for assessing gait is three-dimensional (3D) gait analysis. This requires the accurate placement of small skin-mounted markers on anatomical landmarks on the body. The positions of these markers are then measured using infrared cameras to calculate joint angle graphs (and moments and powers when synchronous force data is captured). This is a highly technical analysis that is time-consuming (with a reported median duration of 80 min for subject preparation, data collection, and physical examination across Europe [2]), requires minimal clothing, can trigger anxiety, and thereby may lead to an unnatural walking pattern [3,4]. These challenges are particularly prevalent in very young children, those with sensory processing difficulties, and children with behavioural problems or intellectual disabilities [3]. Despite the challenges, 3D gait analysis remains clinically valuable for identifying gait deviations in these children. Therefore, identifying methods that reduce the burden of data collection, while maintaining sufficient accuracy, is essential to ensure the continued clinical utility of gait analysis in these groups. Furthermore, even when markers are placed accurately by a highly skilled practitioner, soft tissue artefacts remain an unsolved problem despite numerous solutions developed to reduce inaccuracies [5,6,7]. Bone deformities, and especially torsional deformities, can cause further inaccuracies in marker placement [8].

One potential solution to tackle the limitations associated with participant discomfort and marker placement is to use markerless motion capture technology [9]. These systems typically rely on high-resolution video footage and machine learning algorithms to estimate joint positions and body segment kinematics without the need for skin-mounted markers. A wide range of markerless systems have been developed to investigate human gait in clinical contexts [10]. Some use depth sensors (e.g., Microsoft Kinect [11]), others rely on monocular or multi-view 2D video-based pose estimators (e.g., OpenPose [12], OpenCap [13]), and others, like Theia3D [14], apply deep learning to synchronised multi-camera video data to generate full-body 3D kinematic models. These systems differ in their data inputs, underlying algorithms, and output formats, but share the potential to streamline workflow, reduce participant burden, and improve ecological validity, particularly for populations in which traditional marker use may be difficult or inappropriate.

Theia3D (v2023.1.0.3161, Theia Markerless Inc., Kingston, ON, Canada) was selected for this study due to its availability as a commercial markerless motion capture system within our research setting, and its compatibility with existing laboratory equipment and software for future studies (e.g., force plates, Visual3D). The Theia3D software uses synchronised video data and deep learning techniques to estimate three-dimensional (3D) human pose without the need for skin-mounted markers. While the internal pose estimation algorithm is proprietary and not openly documented, Theia3D markerless technology has demonstrated the ability to produce results that are comparable to the current marker-based method in adult gait studies [14], with differences as high as 11° (hip flexion/extension) and as low as 2.6° (hip abduction/adduction). In addition, this innovation has shown encouraging preliminary results in a cohort of clinical patients, although further tests are recommended before using it for clinical decision-making [15,16,17,18]. The potential benefits of Theia3D markerless technology are particularly relevant in paediatric contexts, where movement variability, sensory sensitivities, and behavioural compliance often limit the quality or feasibility of marker-based assessments. In these cases, markerless systems may offer a less intrusive and more efficient alternative, though further validation in children is needed.

While early work has explored the validity of Theia3D in pediatric gait, the existing body of evidence remains limited. To date, only one study has quantified the difference between synchronous marker and markerless derived gait kinematics in typically developing children, reporting differences as high as 12° (hip rotation) and as low as 2.5° (foot progression), with marker data processed using the Human Body Model [16]. Further comparison between marker-based and markerless systems has been reported in a mixed cohort of adults and children. D’Souza et al. [18] compared Theia3D with the CAST model in healthy individuals and clinical patients aged 8–61 years, demonstrating generally similar gait patterns but significant differences in hip rotation (~9°), knee rotation (~10°), and pelvic tilt (~6°). However, to our knowledge, no studies have directly compared Theia3D with the conventional gait model (CGM), also called the plug-in gait model (PiG), which is currently the most widely used biomechanical model in clinical gait analysis, particularly in the paediatric population [19,20]. The PiG model is suitable for gait analysis in the paediatric population because it uses a small number of markers and has demonstrated reliability of kinematic and kinetic data, indicating that the magnitude of the errors obtained using this model is clinically reasonable [20]. Since younger children exhibit greater gait variability [21,22], the magnitude of differences between models may also vary depending on age. This study addresses these gaps by quantifying the differences in lower limb 3D gait kinematics derived from marker-based (PiG model) and markerless (Theia3D) motion capture for typically developing children and identifying if and how children’s age might affect these differences.

## 2. Materials and Methods

### 2.1. Participants and Data Collection

Ten healthy children (8 male/2 female, mean (SD) age: 8.3 (2.2) years, height: 1.33 (0.18) m, mass: 31.4 (14.2) kg) volunteered to participate in this study at the Liverpool John Moores University Biomechanics laboratory (Liverpool, UK). The following clinical data were collected: age, height, mass, leg length, inter-ASIS distance, and knee and ankle width. The sample included three children aged six years, four children aged eight years, two children aged ten years, and one child aged 13 years. Participants were able to walk independently without assistive devices, were free from musculoskeletal or neurological conditions that affected their gait, and had no previous history of lower limb surgeries.

Ethical approval was obtained from the University Research Ethics Committee (22/SPS/033). Written informed consent was obtained from the legal guardian of each participant, consenting to the use of the child’s data for this healthcare research.

A single mixed-camera system of ten infrared (Oqus 7+, Qualisys AB, Gothenburg, Sweden) and seven colour video cameras (Miqus Video, Resolution: 1920 × 1080p, Qualisys AB, Gothenburg, Sweden) captured all trials at 85 Hz. Whole-system synchronisation was achieved using the Qualisys Precision Time Protocol, with all data, including calibration of the global reference frame, captured within a single instance of Qualisys Track Manager (Qualisys AB, Gothenburg, Sweden). Retro-reflective markers, with a diameter of 14.5 mm, were placed on the lower limb of the participants according to the PiG model [23]. The marker placement was undertaken by a single assessor, whose repeatability is regularly tested according to the CMAS standards.

A recording of the participant standing in a stationary position was captured to define a marker-based model during post-processing. Participants then walked barefoot at self-selected speed on a 10 m walkway. One static and one walking trial were collected and processed to evaluate the knee varus/valgus kinematic profile. If the knee varus/valgus profile exhibited crosstalk with knee flexion, the thigh wand marker was adjusted, and a new static trial was collected [20]. Each participant was then asked to walk until five separate kinematic trials were conducted. This decision aligns with common clinical gait analysis practices, where data collection is typically limited to five trials per condition due to participant fatigue and time constraints, particularly in pediatric populations. While guided by clinical feasibility, this number also aligns with prior research, which reports that five gait cycles demonstrate good kinematic repeatability in children [24].

### 2.2. Data Analysis

**Figure 1:**
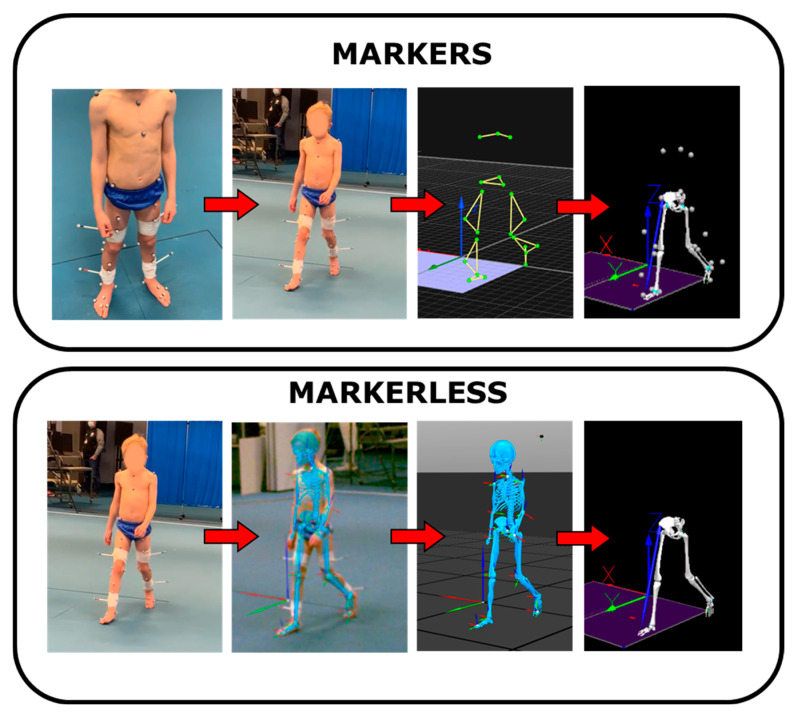
Visual comparison of marker-based and markerless motion capture workflows for a representative participant. **Top row:** (1) Child participant standing with Plug-in Gait (PiG) marker set attached (face blurred for anonymity); (2) walking trial with PiG markers visible; (3) markers labelled within Qualisys Track Manager (QTM); and (4) Visual3D-generated skeletal model based on the marker trajectories. **Bottom row:** (1) child walking; (2) walking trial with Theia3D-generated skeletal overlay; (3) three-dimensional model output from Theia3D; and (4) Visual3D-generated skeletal model based on the markerless joint centre outputs. This figure illustrates the key steps in both data processing pipelines and highlights the differences in model generation between marker-based and markerless systems. Joint centre locations in the Theia3D overlay were determined by proprietary deep learning algorithms and are not explicitly labelled or rendered as visible discrete key points.

Markerless motion capture video data were processed using Theia3D (v2023.1.0.3161, Theia Markerless Inc., Kingston, ON, Canada) to generate the default inverse kinematic full body model with 6 degrees of freedom (dof) at the pelvis, 3 dof at the hip, and 2 dof at the knee and ankle. Theia3D uses a deep convolutional neural network to perform 2D pose estimation on synchronised video frames. This network is trained on a large dataset of human images annotated with anatomical landmarks, enabling the automatic detection of key points across multiple views. The 2D key points extracted from each camera view are then triangulated to reconstruct the 3D locations of anatomical landmarks. These points are used to fit a biomechanical model to the subject’s motion through an optimisation procedure, yielding full-body 3D pose estimations. The system does not rely on physical markers. Instead, it estimates the positions of “virtual markers” by directly identifying anatomical features in the video frames. These virtual markers are consistent with traditional marker-based definitions but are generated automatically based on visual inference [14]. Theia3D outputs that represent the participant’s pose were exported as C3D files to Visual3D (C-Motion, Germantown, MD, USA). Marker-based motion capture data were tracked in QTM, and polynomial interpolation was applied to gaps smaller than 10 frames. The data was then exported for further analysis to Visual3D (Figure 1).

Two skeletal models were created in Visual3D: one, which Visual3D automatically generated when data from Theia3D was loaded, and a second marker-based model, which tracked the marker trajectories, was manually defined. The marker-based and markerless data were filtered using a 4th-order Butterworth filter with a lowpass cut-off frequency of 6 Hz.

Pelvis and lower limb joint angles were calculated using the Visual3D models in the sagittal, coronal, and transverse planes. One left-sided gait cycle was used from each of the five gait trials for analysis. Heel strike gait events were determined using foot marker kinematics, defined as the local maxima in the anterior displacement of the toe relative to the pelvis [25]. They were applied to the markerless data to ensure that the kinematic measurements were compared between the systems for identical gait cycles. Marker-based and markerless data were then time-normalised to 101 points.

### 2.3. Statistical Analysis

The differences between systems were assessed using Generalised Additive Mixed Models (GAMM) [26], fitted to the data to identify the systematic and random effects of measurements by model (PiG, Theia3D), time (100% of the gait cycle), and age. Spline expansions were used to model the observed nonlinearities in the measurements. The selection of spline complexity was automated and based on complexity control criteria.

Approximate F-test statistics were obtained to test the null hypothesis of equality of measurements across model types, controlling for age and repeated measurements within subjects. Significance level was scaled to 0.005 by Bonferroni correction to control the family-wise error rate of repeated tests on correlated signals. The analyses were implemented in the R language using the mgcv, itsadug, and ggplot2 libraries.

Root mean square deviation (RMSD) was calculated for each gait cycle for each participant to compare the pairwise differences in marker and markerless-derived lower limb gait kinematics. This involved computing the square root of the mean of the squared differences between joint angle values at each time-normalised point across the gait cycle. We selected RMSD because it offers a concise and widely accepted summary of deviations between datasets. It is commonly reported in previous validation studies of markerless motion capture systems [14,17,18]. Including RMSD in our analysis not only facilitates direct comparison with those studies but also allows the reader to interpret our results in the context of clinically meaningful error thresholds. The current guideline threshold for inter-assessor repeatability error in clinical gait laboratories in the UK and Northern Ireland is 5–10° [27], and this was used as a guideline when interpreting differences between the PiG and Theia3D models.

## 3. Results

**Figure 2:**
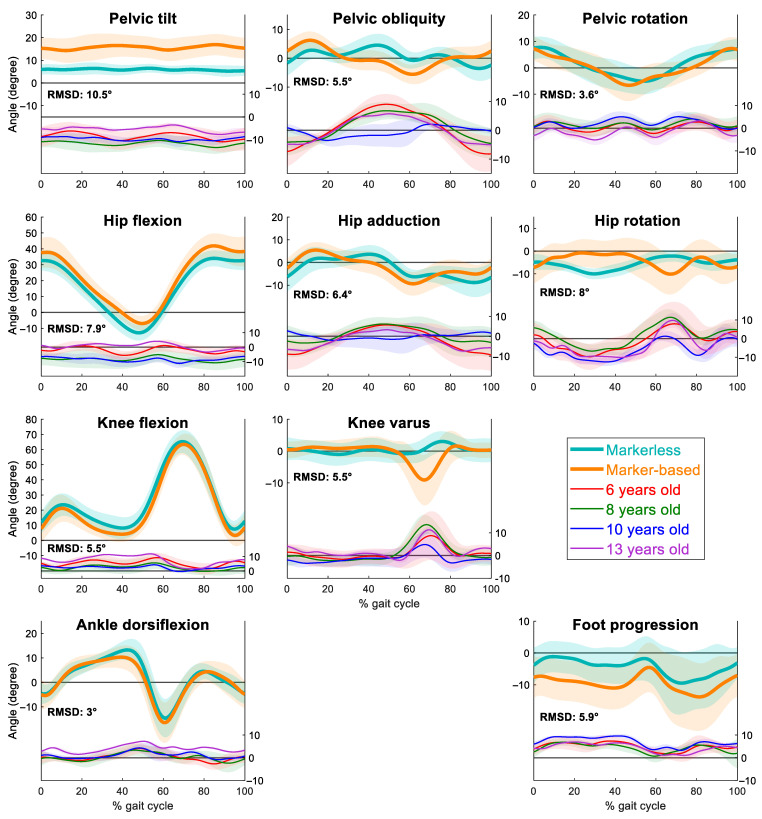
Mean ± standard deviation (SD) joint angles of the pelvis, hip, knee, and ankle graphs in the sagittal, coronal and transverse plane for the left leg measured by the markerless (bold teal with shaded teal areas) and marker-based (bold orange with shaded orange areas) motion capture systems for 10 typically developing children. Shaded areas represent the inter-trial variability (±1 SD) across the cohort. The upper panels show joint angle curves for each system, while the lower panels display the average difference (markerless—marker-based) stratified by age group: 6 years (red), 8 years (green), 10 years (blue), and 13 years (purple). Average pairwise RMSDs between the two systems are inset in each joint angle plot.

There were significant interactions and differences between the Theia3D and PiG model across the gait cycle and between age groups, for most joint angles and planes of motion. Thereby, the null hypothesis of no difference between the Theia3D and PiG model was rejected. The statistical model was able to explain between 73 and 97% of the observed variation across the joint levels (Table 1). The RMSD was less than 5° for pelvic rotation and ankle dorsiflexion/plantarflexion angles (Figure 1). Pelvic tilt had the highest RMSD (10.5°), with the Theia3D model showing less anterior tilt across the gait cycle. Hip rotation had a high RMSD (8.0°), with the Theia3D model showing more external hip rotation during the stance phase and less during the initial swing phase (Figure 2).

Visual inspection of the curves suggests there were comparable patterns and ranges of motion for some, but not all, joints and planes of motion. Pelvic tilt, pelvic obliquity, hip rotation, hip abduction/adduction, foot progression across the entire gait cycle, and knee varus/valgus during the swing phase show the most notable visible differences in gait pattern. Similar gait patterns and/or low RMSD were reported for all other joint levels and planes of motion (Figure 1).

The difference between marker-based and markerless data also depended on age and was especially notable for pelvis tilt, pelvis obliquity, hip and knee flexion/extension, hip abduction/adduction, and hip rotation (Figure 1). Among these, 10-year-olds exhibited the smallest differences in pelvis obliquity and hip abduction/adduction. In contrast, the largest differences in hip flexion/extension and hip rotation were observed in both 8- and 10-year-olds, while the 13-year-olds showed the greatest discrepancies in knee flexion/extension.

## 4. Discussion

This study reports the differences in lower limb 3D gait kinematics derived from marker-based (PiG model) and markerless (Theia3D) motion capture for typically developing children. The markerless system produced significantly different kinematics compared to the PiG model for all joint levels and planes of motion throughout the gait cycle. The magnitude of difference between the PiG and Theia3D gait kinematics also varied by age, with no consistent pattern across joint levels and planes of motion.

Despite the significant differences between models, our RMSD errors are similar or better than previously published results that compare a range of marker-based models to Theia3D in differing age groups of typically developed adults and children [14,18]. Notably, the large differences we report for pelvic tilt and hip rotation are comparable to those previously reported in TD children (pelvic tilt = 9.2°, hip rotation = 12° [17]). Our work builds on previous research by identifying that the child’s age may influence the differences reported between models, a finding that has not been previously reported. The differences between age groups were notable for pelvis obliquity, hip and knee flexion/extension, hip abduction/adduction, and hip rotation (Figure 1); however, there was no clear evidence that one age group consistently differed more than the others. Reduced differences in older children might be explained by a reduction in intra-subject gait variability linked to maturation [21,22], but this explanation does not hold across all joints. While our findings provide preliminary insights into the differences between markerless and marker-based gait data in typically developing children, the small sample size limits the generalizability of our results. As gait characteristics can vary substantially with age, development, and individual variability, caution is warranted in extending these results to broader populations. Future work should aim to include larger and more diverse samples to characterise these differences across developmental stages.

Although the marker-based knee varus/valgus gait curves were assessed for crosstalk and fell under 10°, the presence of the knee valgus wave during the swing phase indicates the possibility of kinematic crosstalk. Despite this, the marker-based hip rotation aligned around neutral in the stance phase, whereas the markerless data indicates mild external rotation. Despite being a well-known challenge to accurately assess hip rotation with the marker-based system, the accuracy of the markerless system in capturing hip rotation in children remains unknown. Therefore, the difference may be due to a marker placement error in the marker-based data, systemic errors in the markerless system, or both, resulting in a higher overall RMSD.

Some of the differences between the PiG and Theia3D models are likely explained by differences in the underlying segment definitions. For example, in the sagittal plane, the PiG-defined pelvis is moderately offset to anterior tilt, whereas the Theia3D model appears to generate a more neutral pelvis. This led to a systematic offset in our analysis and is a common feature of existing validation studies that compared different marker-based models [14,16,17,18,28]. Errors in joint centre position estimation may also contribute to further errors in lower limb joint kinematics [29]. The PiG model has been reported to produce larger mean deviations, especially in the medial-lateral direction, compared to other hip joint centre equations [29,30]. This may explain the differences in hip abduction/adduction, especially during the early stance and swing phase. More broadly, both marker-based and markerless motion capture systems are subject to methodological challenges such as occlusion and errors in identifying body segment positions. Marker-based approaches are particularly affected by soft tissue artefacts and mislabeling of markers, while markerless systems may encounter difficulties if clothing obscures anatomical landmarks. However, previous findings suggest that the choice of clothing across multiple sessions does not substantially increase variation [31]. Although multiple camera views can reduce the impact of occlusion for both systems, neither approach is immune to these sources of error. The influence of soft tissue movement on markerless motion capture remains underexplored and warrants further investigation. Markerless kinematics generated by Theia3D may be affected by several factors related to the training dataset it learns from, including the age, sex, and ethnicity of the individuals included. It is also unlikely that the underlying artificial neural network of Theia3D was trained on children with markers attached to their legs. Any implicit bias within the training dataset could result in propagated errors in the data; however, since Theia3D is a proprietary system, researchers have limited control over how the machine learning models process the data.

## 5. Conclusions

This study investigated the differences in lower limb kinematics between markerless (Theia3D) and the commonly used marker-based PiG model in typically developed children. The results showed significant differences across all kinematics, which varied with age; however, the RMSD errors in this study were comparable to those previously published. The differences between models emphasise that the two systems cannot be used interchangeably at this stage. Further investigations are needed to determine whether markerless lower limb kinematics are an accurate method in typically developing children, particularly in clinical populations.

## Figures and Tables

**Table 1:** Statistical differences between Theia3D and PiG.

	Signal	Nonlinear Trend over Time	Nonlinear Trend over Age	Interaction Between Age and Time	R^2^
Pelvis	Tilt	F(8.45, 9954.67) = 61.02	F(2.99, 9954.67) = 899.35	F(8.35, 9954.67) = 12.35	0.91
		*p* < 0.001	*p* < 0.001	*p* < 0.001	
	Obliquity	F(8.61, 9949.58) = 964.12	F(2.99, 9949.58) = 119.92	F(8.95, 9949.58) = 340.67	0.78
		*p* < 0.001	*p* < 0.001	*p* < 0.001	
	Rotation	F(8.85, 9954.26) = 68.07	F(2.97, 9954.26) = 129.16	F(8.07, 9954.26) = 13.04	0.82
		*p* < 0.001	*p* < 0.001	*p* < 0.001	
Hip	Flex/Ext	F(8.60, 9949.56) = 41.07	F(2.99, 9949.56) = 939.66	F(8.35, 9949.56) = 11.58	0.97
		*p* < 0.001	*p* < 0.001	*p* < 0.001	
	Abd/	F(8.34, 9952.34) = 340.36	F(2.98, 9952.34) = 147.27	F(8.88, 9952.34) = 153.28	0.78
	Adduction	*p* < 0.001	*p* < 0.001	*p* < 0.001	
	Int/ Ext	F(8.93, 9952.22) = 716.78	F(3.00, 9952.22) = 700.88	F(8.74, 9952.22) = 17.41	0.74
	Rotation	*p* < 0.001	*p* < 0.001	*p* < 0.001	
Knee	Flex/Ext	F(8.60, 9942.91) = 47.45	F(2.967, 9942.91) = 176.63	F(8.27, 9942.91) = 8.07	0.96
		*p* < 0.001	*p* < 0.001	*p* < 0.001	
	Varus/Valgus	F(8.96, 9949.27) = 579.42	F(2.99, 9949.27) = 184.13	F(8.91, 9949.27) = 77.50	0.73
		*p* < 0.001	*p* < 0.001	*p* < 0.001	
Ankle	Plantar/	F(8.42, 9951.03) = 66.90	F(2.82, 9951.03) = 109.02	F(4.26, 9951.03) = 3.12	0.89
	Dorsiflexion	*p* < 0.001	*p* < 0.001	*p* = 0.006	
	Foot	F(8.59, 9947.86) = 89.44	F(2.99, 9947.86) = 96.36	F(7.22, 9947.86) = 3.01	0.87
	Progression	*p* < 0.001	*p* < 0.001	*p* = 0.002	

## Data Availability

The dataset is available on request from the authors. The raw data supporting the conclusions of this article will be made available by the authors on request.

## References

[1] Perry J., Burnfield J. (2010). Gait Analysis: Normal and Pathological Function.

[2] Armand S., Sawacha Z., Goudriaan M., Horsak B., van der Krogt M., Huenaerts C., Daly C., Kranzl A., Boehm H., Petrarca M. (2024). Current practices in clinical gait analysis in Europe: A comprehensive survey-based study from the European society for movement analysis in adults and children (ESMAC) standard initiative. Gait Posture.

[3] Hallemans A., Van de Walle P., Wyers L., Verheyen K., Schoonjans A.-S., Desloovere K., Ceulemans B. (2019). Clinical usefulness and challenges of instrumented motion analysis in patients with intellectual disabilities. Gait Posture.

[4] Hulleck A.A., Mohan D.M., Abdallah N., El Rich M., Khalaf K. (2022). Present and future of gait assessment in clinical practice: Towards the application of novel trends and technologies. Front. Med. Technol..

[5] Leboeuf F., Barre A., Aminian K., Sangeux M. (2023). On the accuracy of the Conventional gait Model: Distinction between marker misplacement and soft tissue artefact errors. J. Biomech..

[6] Fonseca M., Gasparutto X., Leboeuf F., Dumas R., Armand S. (2020). Impact of knee marker misplacement on gait kinematics of children with cerebral palsy using the Conventional Gait Model-A sensitivity study. PLoS ONE.

[7] McGinley J.L., Baker R., Wolfe R., Morris M.E. (2009). The reliability of three-dimensional kinematic gait measurements: A systematic review. Gait Posture.

[8] Passmore E., Graham H.K., Sangeux M. (2018). Defining the medial-lateral axis of the femur: Medical imaging, conventional and functional calibration methods lead to differences in hip rotation kinematics for children with torsional deformities. J. Biomech..

[9] Colyer S.L., Evans M., Cosker D.P., Salo A.I.T. (2018). A review of the evolution of vision-based motion analysis and the integration of advanced computer vision methods towards developing a markerless system. Sports Med. Open.

[10] Wade L., Needham L., McGuigan P., Bilzon J. (2022). Applications and limitations of current markerless motion capture methods for clinical gait biomechanics. PeerJ.

[11] Mentiplay B.F., Perraton L.G., Bower K.J., Pua Y.-H., McGaw R., Heywood S., Clark R.A. (2015). Gait assessment using the Microsoft Xbox One Kinect: Concurrent validity and inter-day reliability of spatiotemporal and kinematic variables. J. Biomech..

[12] Cao Z., Simon T., Wei S.E., Sheikh Y. Realtime multi-person 2d pose estimation using part affinity fields. Proceedings of the IEEE Conference on Computer Vision and Pattern Recognition 2017.

[13] Uhlrich S.D., Falisse A., Kidziński Ł., Muccini J., Ko M., Chaudhari A.S., Hicks J.L., Delp S.L. (2023). OpenCap: Human movement dynamics from smartphone videos. PLOS Comput. Biol..

[14] Kanko R.M., Laende E.K., Davis E.M., Selbie W.S., Deluzio K.J. (2021). Concurrent assessment of gait kinematics using marker-based and markerless motion capture. J. Biomech..

[15] Wren T.A.L., Isakov P., Rethlefsen S.A. (2023). Comparison of kinematics between Theia markerless and conventional marker-based gait analysis in clinical patients. Gait Posture.

[16] McGuirk T.E., Perry E.S., Sihanath W.B., Riazati S., Patten C. (2022). Feasibility of markerless motion capture for three-dimensional gait assessment in community settings. Front. Hum. Neurosci..

[17] Wishaupt K., Schallig W., van Dorst M.H., Buizer A.I., van der Krogt M.M. (2024). The applicability of markerless motion capture for clinical gait analysis in children with cerebral palsy. Sci. Rep..

[18] D'Souza S., Siebert T., Fohanno V. (2024). A comparison of lower body gait kinematics and kinetics between Theia3D markerless and marker-based models in healthy subjects and clinical patients. Sci. Rep..

[19] Flux E., van der Krogt M.M., Cappa P., Petrarca M., Desloovere K., Harlaar J. (2020). The Human Body Model versus conventional gait models for kinematic gait analysis in children with cerebral palsy. Hum. Mov. Sci..

[20] Kainz H., Graham D., Edwards J., Walsh H.P.J., Maine S., Boyd R.N., Lloyd D.G., Modenese L., Carty C.P. (2017). Reliability of four models for clinical gait analysis. Gait Posture.

[21] Sutherland D. (1997). The development of mature gait. Gait Posture.

[22] Oudenhoven L.M., Booth A.T.C., Buizer A.I., Harlaar J., van der Krogt M.M. (2019). How normal is normal: Consequences of stride to stride variability, treadmill walking and age when using normative paediatric gait data. Gait Posture.

[23] Davis R.B., Ounpuu S., Tyburski D., Gage J.R. (1991). A gait analysis data collection and reduction technique. Hum. Mov. Sci..

[24] Steinwender G., Saraph V., Scheiber S., Zwick E.B., Uitz C., Hackl K. (2000). Intrasubject repeatability of gait analysis data in normal and spastic children. Clin. Biomech..

[25] Zeni J.A., Richards J.G., Higginson J.S. (2008). Two simple methods for determining gait events during treadmill and overground walking using kinematic data. Gait Posture.

[26] Wood S.N. (2017). Generalized Additive Models: An Introduction with R.

[27] Stewart C., Eve L., Durham S., Holmes G., Stebbins J., Harrington M., Corbett M., Kiernan D., Kidgell V., Jarvis S. (2023). Clinical movement analysis society–UK and Ireland: Clinical movement analysis standards. Gait Posture.

[28] Kanko R.M., Outerleys J.B., Laende E.K., Selbie W.S., Deluzio K.J. (2024). Comparison of Concurrent and Asynchronous Running Kinematics and Kinetics from Marker-Based and Markerless Motion Capture Under Varying Clothing Conditions. J. Appl. Biomech..

[29] Leboeuf F., Reay J., Jones R., Sangeux M. (2019). The effect on conventional gait model kinematics and kinetics of hip joint centre equations in adult healthy gait. J. Biomech..

[30] Mantovani G., Ng K.C.G., Lamontagne M. (2016). Regression models to predict hip joint centers in pathological hip population. Gait Posture.

[31] Augustine S., Foster R., Barton G., Lake M.J., Sharir R., Robinson M.A. (2025). The inter-trial and inter-session reliability of Theia3D-derived markerless gait analysis in tight versus loose clothing. PeerJ.

